# The correlation and prognostic value of serum levels of soluble programmed death protein 1 (sPD-1) and soluble programmed death-ligand 1 (sPD-L1) in patients with hepatocellular carcinoma

**DOI:** 10.1007/s00262-018-2271-4

**Published:** 2018-12-01

**Authors:** Boyang Chang, Tao Huang, Huajun Wei, Lujun Shen, Duo Zhu, Wenjun He, Qifeng Chen, Huihua Zhang, Yunjian Li, Ruopan Huang, Wang Li, Peihong Wu

**Affiliations:** 1 Department of Vascular Interventional Radiology, The Third Affiliated Hospital of Sun Yat-sen University, Guangzhou, 510630 Guangdong People’s Republic of China; 2 State Key Laboratory of Oncology in South China, Collaborative Innovation Center for Cancer Medicine, Sun Yat-sen University Cancer Center, Guangzhou, 510060 Guangdong People’s Republic of China; 3 Department of Medical Imaging and Interventional Radiology, Sun Yat-sen University Cancer Center, 651 Dongfeng East Road, Guangzhou, 510060 Guangdong People’s Republic of China; 4 Department of Medical Oncology, Gaozhou People’s Hospital, Gaozhou, 525200 Guangdong People’s Republic of China; 5 Department of Medical Statistic and Epidemiology, School of Public Health, Sun Yat-sen University, Guangzhou, 510080 Guangdong People’s Republic of China; 6 RayBiotech, Inc, Guangzhou, 510600 Guangdong People’s Republic of China; 7 South China Biochip Research Center, Guangzhou, 510600 Guangdong People’s Republic of China; 8 RayBiotech, Inc, Norcross, GA 30092 USA

**Keywords:** Inflammatory cytokines, Immune checkpoint, Immunohistochemistry, Tumor-infiltrating lymphocytes

## Abstract

**Background:**

Blocking the programmed death protein 1 (PD-1)/programmed death-ligand 1 (PD-L1) pathway in hepatocellular carcinoma (HCC) is a very promising approach in immunotherapy. However, the correlation and prognostic values of serum soluble PD-1 and PD-L1 (sPD-1/sPD-L1) have not been explored conjointly in HCC patients.

**Methods:**

This study retrospectively included 120 HCC patients receiving radical resection. The serum levels of sPD-1/sPD-L1 and inflammatory cytokines were measured by antibody array assay. Immunohistochemistry was applied to assess both the expression of membrane-bound PD-L1, and the number of CD4^+^ tumor-infiltrating lymphocytes (TILs) and CD8^+^ TILs.

**Results:**

The best cut-off values of sPD-1 and sPD-L1 for predicting disease-free survival (DFS) were 33.0 µg/ml and 11.2 µg/ml, respectively. Multivariable analysis showed that sPD-L1 was a negative independent prognostic factor [DFS, Hazard Ratio (HR) 2.58, 95% CI 1.14–5.84, *P* = 0.023; overall survival (OS), HR 1.77, 95% CI 1.01–3.12, *P* = 0.048], while sPD-1 was a favorable independent prognostic factor (DFS, HR 0.32, 95% CI 0.14–0.74, *P* = 0.007; OS, HR 0.54, 95% CI 0.30–0.98, *P* = 0.044) in HCC patients. We also observed some similar associations between inflammatory cytokines (IL-10, IL-17, TNF-α) and sPD-1 or sPD-L1, as well as a close positive association between sPD-1 and sPD-L1. No significant associations of sPD-1/sPD-L1 with either intra-tumoral PD-L1 expression, or the numbers of CD4^+^ TILs and CD8^+^ TILs were determined.

**Conclusions:**

Our findings indicate that sPD-1 and sPD-L1 are independent prognostic factors with opposite prognostic roles in predicting both DFS and OS in HCC patients.

**Electronic supplementary material:**

The online version of this article (10.1007/s00262-018-2271-4) contains supplementary material, which is available to authorized users.

## Introduction

Hepatocellular carcinoma (HCC) is the 6th most common cancer and the second leading cause of cancer-related deaths worldwide [1]. Although the efficacy of HCC treatment has significantly improved over the last decade due to the progress in resection criteria and locoregional treatment techniques, more than half of early HCC patients will suffer from recurrence within 5 years after resection; the majority of recurrences develop within the first year after resection [2].

Increasing evidence suggests that cancer immune suppression and immune escape play essential roles in tumor progression. Among these processes, the activation of the programmed death protein 1/programmed death ligand 1 (PD-1/PD-L1) pathway was identified as the most critical mechanism of tumor evasion, inhibiting T-cell proliferation, inducing T-cell exhaustion and enhancing the activity of regulatory T cells [3].

The expression of membrane-bound PD-L1 (mPD-L1) is associated with the prognosis of several types of malignant tumors [4]. However, the prognostic significance of mPD-L1 expression in liver cancer remains controversial, and further studies are needed to address the problem [5–8].

In addition to the membrane-bound forms, soluble PD-1/PD-L1 (sPD-1/sPD-L1) recently have recently been detected in the blood of cancer patients [9, 10]. A few clinical studies have evaluated their prognostic values in patients with cancer and explored the associations of sPD-1/sPD-L1 levels with clinicopathological factors [11–14]. In terms of HCC, Finkelmeier et al. [14] found that patients with an increased level of sPD-L1 had an elevated mortality risk. However, in their study, the association and prognostic value of sPD-1 and sPD-L1 were not explored conjointly; furthermore, the influence of sPD-1/sPD-L1 levels on disease-free survival was not investigated. Therefore, additional studies are needed to explore the potential prognostic value of sPD-1/sPD-L1 in HCC.

In the present study, we aimed to investigate the associations between sPD-1/sPD-L1 and clinicopathological factors and assess their influence on survival in HCC patients. In addition, we analyzed the prognostic value of mPD-L1, and its association with sPD-1/sPD-L1.

## Materials and methods

### Patient selection

Between March 2008 and December 2014, the medical records of consecutive patients treated for HCC by the same medical team at Sun Yat-sen University Cancer Center were reviewed. The inclusion criteria were: (1) pathologically diagnosed HCC; and (2) curative surgical resection as the initial treatment. The exclusion criteria were: (1) previous or concurrent cancer; (2) active infections other than chronic hepatitis B virus; (3) history of organ allograft; (4) pregnancy or breastfeeding; and (5) lack of tumor tissue samples; (6) lack of preoperative serum samples. Ultimately, a total of 120 patients were included in the study.

Clinicopathological characteristic including age, gender, Child-Pugh score, Barcelona clinic liver cancer (BCLC) stage, hepatitis B virus (HBV) history, HBV DNA level, alpha-fetoprotein (AFP) level, C-reactive protein (CRP) level, aspartate aminotransferase (AST) level, alanine aminotransferase (ALT) level, gamma-glutamyl transpeptidase (GGT) level, tumor size, tumor number, grades of differentiation, capsular invasion and microscopic vascular invasion were obtained from the electronic medical record.

### Antibody array assay

We collected 120 preoperative serum samples from the specimen bank of our hospital. All the samples were analyzed using human immune checkpoint molecule array that contained PD-1 and PD-L1 (RayBiotech, Norcross, GA, USA, QAH-ICM-1-1) and a custom-made in-house array (RayBiotech) that contained IFN-γ, TNF-α, IL-2, IL-4, IL-6, IL-10, IL-12, IL-15, IL-16, and IL-17. All experiments were conducted according to the manufacturer’s instructions. Briefly, after 60 min of incubation with blocking buffer, 60 µL of twofold-diluted serum samples was added to each well. After overnight incubation at 4 °C and extensive washing, the biotin-labeled detection antibody was added for 2 h and then washed away. Alexa Fluor 555-conjugated streptavidin was then added and incubated for 1 h at room temperature. The signals (532 nm excitation) were scanned and extracted using an InnoScan 300 scanner (Innopsys, Carbonne, France). Raw data from the array scanner were provided as images (.tif files) and spot intensities (tab-delimited.txt file) through Mapix 7.3.1 software. Individual array spots were locally background subtracted and normalized against two positive controls. The mean signal-background for each set of duplicate standards and samples was calculated. Then, the standard curve was plotted on log–log graph paper, with a standard concentration on the *x*-axis and signal-background on the *y*-axis. Finally, the best-fit straight line was drawn through the standard points. The concentrations of all serum proteins detected were determined according to their respective standard curve.

### Immunohistochemical staining

All of the 120 patient tumor tissue samples were analyzed immunohistochemically. Immunohistochemistry (IHC) was performed on 5 mm sections of formalin-fixed, paraffin-embedded tissue samples. The paraffin sections were deparaffinized with xylene and rehydrated in alcohol. Antigen retrieval was accomplished by boiling citrate buffer and endogenous peroxidase activity was blocked with 3% H_2_O_2_ followed by staining with anti-PD-L1 antibody (1:100; Cell Signaling Technology, Danvers, MA, USA), anti-CD4 antibody (1:500; ZSGB-BIO, Beijing, China) or anti-CD8 antibody (1:500; ZSGB-BIO, Beijing, China) overnight at 4 °C. After washing, the sections were processed with a MaxVision^TM^ HRP-Polymer anti-Rabbit IHC Kit at room temperature (Maixin, Fuzhou, China) and then developed with a DAB Horseradish Peroxidase Color Development Kit (Maixin, Fuzhou, China) and counterstained with hematoxylin.

### Evaluation of PD-L1 expression on tumor cells and the number of tumor-infiltrating lymphocytes in HCC

The degree of immunostaining was scored independently by two observers. The interrater reliability (Fleiss’ kappa value) for each IHC staining factor between the two observers is shown in Supplementary Table 1. The proportions of PD-L1 positive cells among the total tumor cells were classified according to the following percentages: ≥ 5%, 1–5%, and < 1%; the tumor positivity was defined using the cutoff of 5%, which was based on a previous study [15].

The numbers of CD4^+^ tumor-infiltrating lymphocytes (TILs) and CD8^+^ TILs were calculated by counting their total number in 10 independent high-power (× 200) microscopic fields for each tissue sample; a scale of 0–9 was used based on the number of positively stained cells: 0 = 0–10, 1 = 11–20, 2 = 21–30, 3 = 31–40, 4 = 41–50, 5 = 51–60, 6 = 61–70, 7 = 71–80, 8 = 81–90, 9 = 91–max [16]. The median of 4 was used as the cutoff for both biomarkers.

To assess the number of Foxp3^+^ TILs, a scale of 0–3 was used based on the percentage of positive lymphocyte cells: 0 (no staining), 1 (0–10%), 2 (10–32%), 3 (> 33%). We utilized 10% as the cutoff according to our previous study [17].

### Follow-up

All HCC patients underwent regular follow-ups at our hospital every 3 months for the first 2 years, every 6 months in years 3–5, and annually after that. The primary endpoints were overall survival (OS) and disease-free survival (DFS). OS was calculated from the date of surgery to either the date of death or the last follow-up. DFS was defined as the time from surgery to the time of recurrence (local or distant) or the date of the last follow-up.

### Statistical methods

The interrater reliability was calculated using Fleiss’ kappa statistics. Statistical comparisons of categorical data were carried out with the Pearson Chi-square test or Fisher’s exact test (if frequencies < 5). The correlation coefficient between different categorical variables was calculated using the Pearson contingency coefficient. Correlation analyses of continuous variables were performed using Spearman’s correlation coefficient analysis. The nonparametric Wilcoxon–Mann–Whitney test was used to compare the results of antibody array assays between different patient cohorts. X-tile 3.6.1 software program (Yale University, New Haven, CT, USA) was used to determine the optimal cut-off values for sPD-1 and sPD-L1 based on the association with OS and DFS. OS and DFS were estimated using the Kaplan–Meier method and compared using the log-rank test. The backward method of the multivariable Cox regression model for OS and DFS was utilized to determine the independent prognostic factors. *P* < 0.05 was considered to indicate statistical significance in all of the analyse. All of the data were analyzed with SPSS 24.0 and GraphPad 5.0 software.

The authenticity of the data has been validated by uploading the critical raw data onto the Research Data Deposit public platform (http://www.researchdata.org.cn), with Research Data Deposit approval number RDDB2018000257.

## Results

### Patient characteristics

By the end of the follow-up period, 59 (49.2%) patients had relapsed, and 27 (22.5%) patients had died of cancer-related causes. The median DFS and OS times for the whole population were 43.8 months (95% CI 41.9–54.0 months) and 65.4 months (95% CI 58.4–68.4 months), respectively. The baseline data for the population are described in Table 1.

**Table 1:** Relationship of clinical factors with serum soluble PD-1 and PD-L1 levels in HCC patients

Variables	All	sPD-1		*P* value	sPD-L1		*P* value
		Low	High		Low	High	
Sex				0.426			0.404^a^
Male	105 (87.5)	25 (83.3)	80 (88.9)		73 (85.9)	32 (91.4)	
Female	15 (12.5)	5 (16.7)	10 (11.1)		12 (14.1)	3 (8.6)	
Age				0.382			0.728
< 50	76 (63.3)	21 (70.0)	55 (61.1)		53 (62.4)	23 (65.7)	
≥ 50	44 (36.7)	9 (30.0)	35 (38.9)		32 (37.6)	12 (34.3)	
BCLC stage				0.235			0.609^a^
A	107 (89.2)	25 (83.3)	82 (91.1)		75 (88.2)	32 (91.4)	
B	13 (10.8)	5 (16.7)	8 (8.9)		10 (11.8)	3 (8.6)	
HBV history				0.312^a^			0.766
No	19 (15.8)	3 (10.0)	16 (17.8)		14 (16.5)	5 (14.3)	
Yes	101 (84.2)	27 (90.0)	74 (82.2)		71 (83.5)	30 (85.7)	
Child-Pugh score				0.562^a^			0.519^a^
5	119 (99.2)	30 (100.0)	89 (98.9)		84 (98.8)	35 (100.0)	
6	1 (0.8)	0 (0.0)	1 (1.1)		1 (1.2)	0 (0.0)	
AFP (ng/mL)				0.111			0.152
≤ 25	53 (44.2)	17 (56.7)	36 (40.0)		34 (40.0)	19 (54.0)	
> 25	67 (55.8)	13 (43.3)	54 (60.0)		51 (60.0)	16 (45.7)	
Tumor size				0.912			0.062
≤ 5 cm	77 (64.2)	19 (63.3)	58 (64.4)		59 (69.4)	18 (51.4)	
> 5 cm	43 (35.8)	11 (36.7)	32 (35.6)		26 (30.6)	17 (48.6)	
Tumor number				0.703^a^			0.952^a^
Single	110 (91.7)	27 (90.0)	83 (92.2)		78 (91.8)	32 (91.4)	
Multiple	10 (8.3)	3 (10.0)	7 (7.8)		7 (8.2)	3 (8.6)	

The data are the numbers of patients, with percentages in parentheses

^a^Fisher’s exact test

### sPD-1 and sPD-L1 levels in HCC patients

**Fig. 1:**
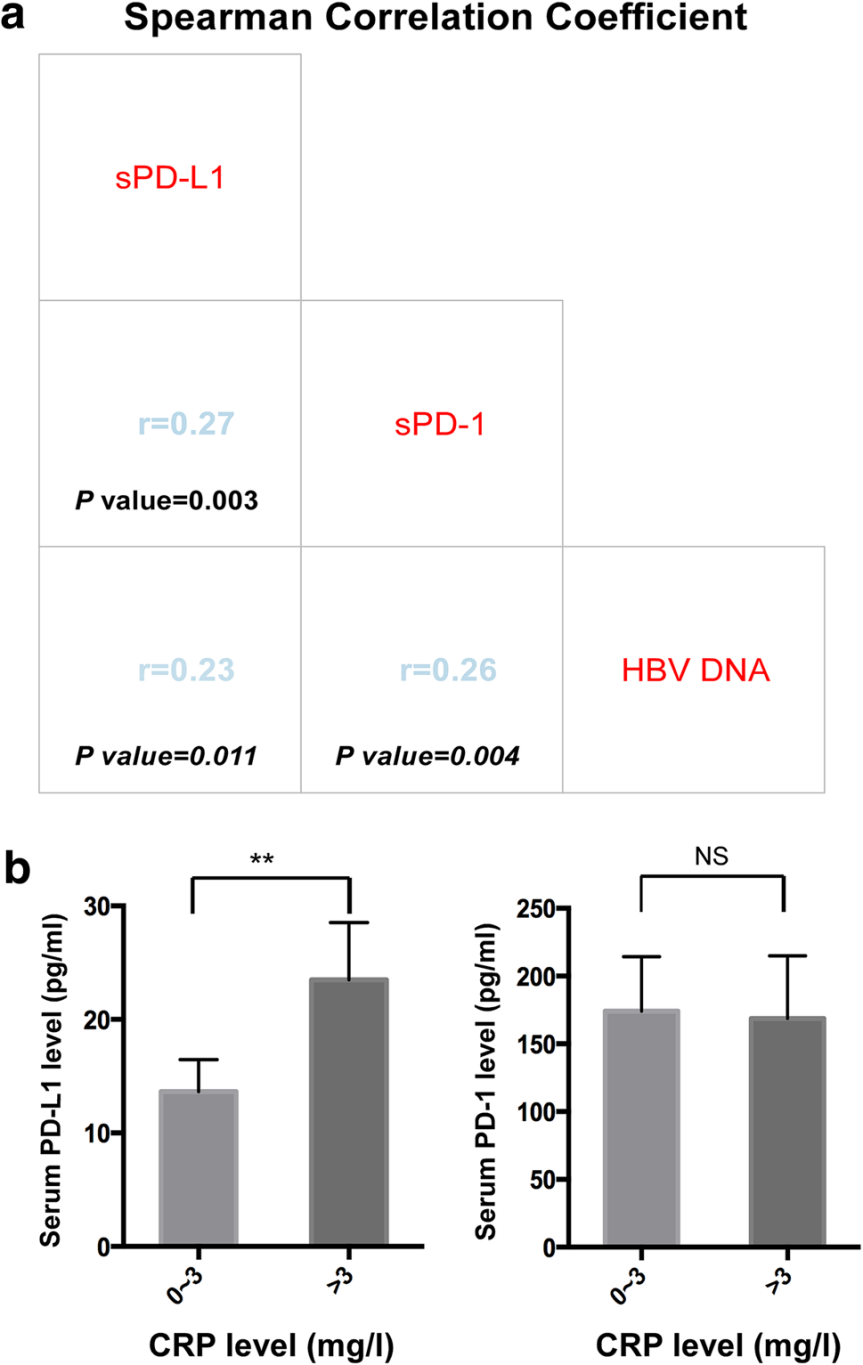
Correlation of sPD-1, sPD-L1 with HBV DNA levels, and their distribution in different CRP levels. Correlation between sPD-1, sPD-L1and HBV DNA levels in the hepatocellular carcinoma patients (**a**). Distribution of sPD-L1 (left) and sPD-1 (right) levels in patients with low vs high CRP (**b**)

The serum level of sPD-1 was detectable in all patients, while that of sPD-L1 was below the lower detection limit in 34 (28.3%) cases. The median values for sPD-1 and sPD-L1 were 82.7 (range 7.6–2886.8 µg/mL) and 5.2 µg/mL (range 0.1–130.0 µg/mL), respectively. We observed that the level of sPD-L1 positively correlated with the level of sPD-1(*r* = 0.27, *P* = 0.003, Fig. 1a). This correlation may suggest a common source of the two soluble checkpoint molecules.

### Associations of serum sPD-1/sPD-L1 levels with prognosis in HCC patients

**Fig. 2:**
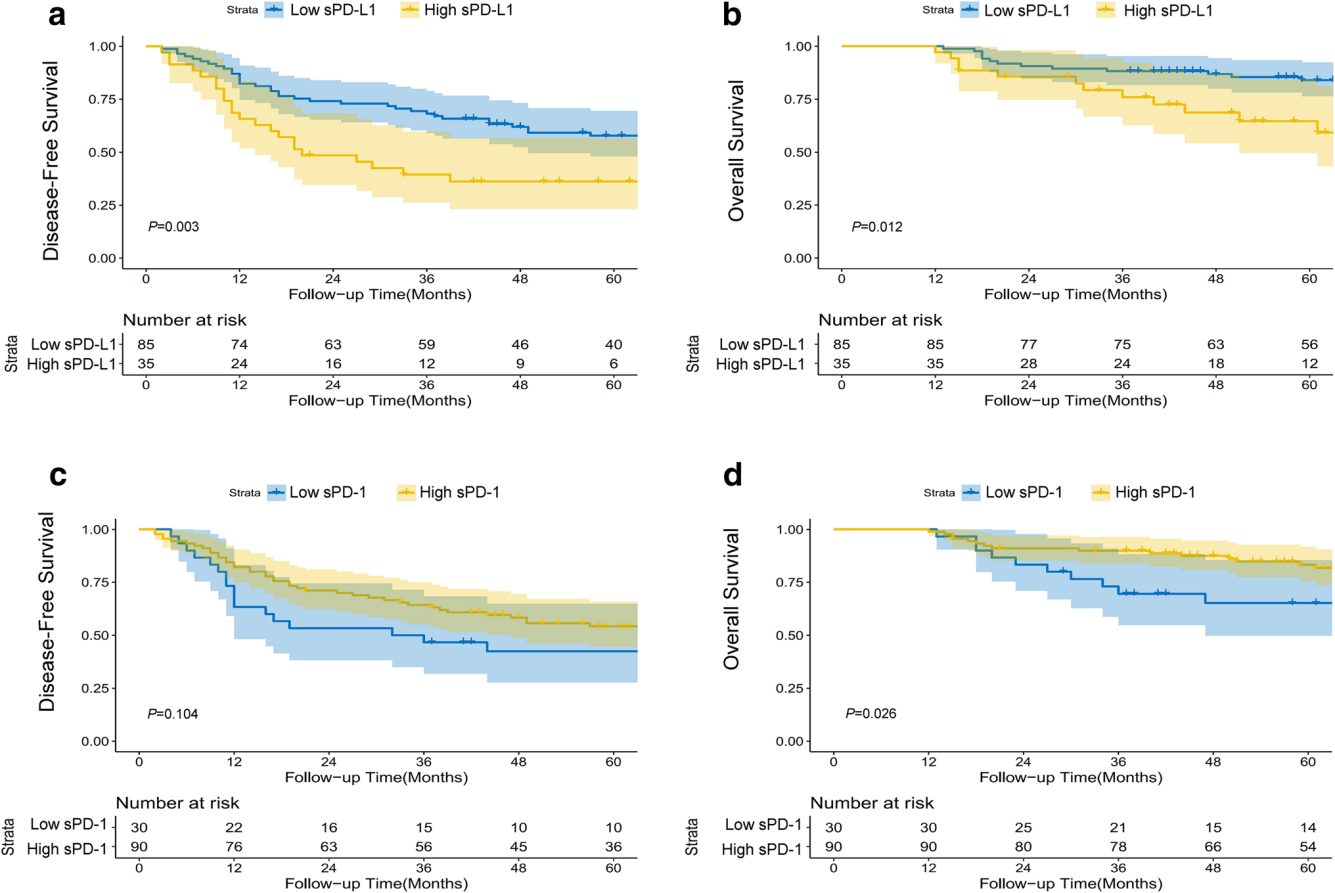
The effects of the levels of sPD-L1 and sPD-1 on prognosis. Disease-free survival and overall survival for patients with high vs low levels of sPD-L1 (**a, b**) and sPD-1 (**c, d**), with the number of patients at risk and 95% confidence intervals

To date, there is no consensus regarding the cut-off values for sPD-1/sPD-L1 in predicting the prognosis of patients with HCC. In our study, X-tile software was applied to determine the best cutoff value for these two continuous variables for predicting DFS. The best cutoff values identified by X-tile for sPD-L1 and sPD-1 were 11.2 µg/mL and 33.0 µg/mL, respectively. In Kaplan–Meier survival analysis, patients with an elevated level of sPD-L1 (> 11.2 µg/mL) had a significantly shorter DFS (Fig. 2a, *P* = 0.003) and OS (Fig. 2b, *P* = 0.012) than patients with a low level of sPD-L1; in contrast, a high level of sPD-1 correlated with a favorable OS (Fig. 2d, *P* = 0.026), as well as a trend toward prolonged DFS (Fig. 2c, *P* = 0.104). Regarding the prognostic value of CD8^+^ TILs, a high number of CD8^+^ TILs correlated with a significantly longer OS (Supplementary Fig. 1c, *P* = 0.021), as well as a tendency toward prolonged DFS (Supplementary Fig. 1d, *P* = 0.090). Multivariable analysis using a Cox regression model suggested that sPD-L1, sPD-1, the level of HBV DNA and intra-tumoral CD8^+^ TILs were independent prognostic factors for DFS (Table 2); while sPD-L1, sPD-1, the level of HBV DNA and microscopic vascular invasion were independent prognostic factors for OS (Table 2).

**Table 2:** Univariable and multivariable analyses of DFS and OS in the population

Variable	Univariable analysis		Multivariable analysis	
	HR (95% CI)	*P* value	HR (95% CI)	*P* value
Disease-free survival				
Gender (female vs male)	0.25 (0.03–1.86)	0.177	–	–
Age (≥ 50 vs < 50)	0.58 (0.25–1.38)	0.219	–	–
BCLC stage (B vs A)	1.08 (0.33–3.60)	0.895	–	–
AFP (> 25 vs ≤ 25)	1.15 (0.54–2.48)	0.718	–	–
HBV DNA (> 10^4^ vs 10^2^–10^4^ vs 0–10^2^)	2.02 (1.17–3.49)	0.011	2.34 (1.28–4.27)	0.006
Tumor size (> 5 cm vs ≤ 5 cm)	1.65 (0.77–3.52)	0.059	–	–
Tumor number (multiple vs single)	0.87 (0.21–3.65)	0.844	–	–
Microscopic vascular invasion (present vs absent)	2.49 (0.86–7.27)	0.094	–	–
Tumoral PD-L1 expression (high vs low)	0.36 (0.11–1.20)	0.097	–	–
CD4^+^ TILs (high vs low)	0.60 (0.27–1.34)	0.216	–	–
CD8^+^ TILs (high vs low)	0.36 (0.15–0.89)	0.026	0.31 (0.11–0.85)	0.022
sPD-L1 (high vs low)	2.60 (1.20–5.62)	0.048	2.58 (1.14–5.84)	0.023
sPD1 (high vs low)	0.43 (0.20–0.93)	0.031	0.32 (0.14–0.74)	0.007
Overall survival				
Gender (female vs male)	0.60 (0.24–1.50)	0.271	–	–
Age (≥ 50 vs < 50)	0.80 (0.47–1.38)	0.426	–	–
BCLC stage (B vs A)	1.33 (0.63–2.79)	0.460	–	–
AFP (> 25 vs ≤ 25)	1.10 (0.66–1.84)	0.717	–	–
HBV DNA (> 10^4^ vs 10^2^–10^4^ vs 0–10^2^)	1.77 (1.26–2.48)	0.001	1.83 (1.29–2.58)	0.001
Tumor size (> 5 cm vs ≤ 5 cm)	1.49 (0.88–2.50)	0.136	–	–
Tumor number (multiple vs single)	1.22 (0.52–2.84)	0.645	–	–
Microscopic vascular invasion (present vs absent)	4.14 (2.08–8.27)	< 0.001	4.37 (2.06–13.68)	< 0.001
Tumoral PD-L1 expression (high vs low)	0.81 (0.44–1.49)	0.492	–	–
CD4^+^ TILs (high vs low)	0.93 (0.55–1.55)	0.768	–	–
CD8^+^ TILs (high vs low)	0.63 (0.37–1.08)	0.093	–	–
sPD-L1 (high vs low)	2.18 (1.29–3.70)	0.004	1.77 (1.01–3.12)	0.048
sPD1 (high vs low)	0.69 (0.45–1.11)	0.104	0.54 (0.30–0.98)	0.044

### Associations of sPD-L1 and sPD-1 level with HBV DNA and inflammatory factors

In Asia, most cases of HCC are driven by chronic HBV infection, thus inflammation is a critical step in HCC initiation and progression. We hypothesized that sPD-1/sPD-L1 might be upregulated in the persistent inflammatory environment triggered by the presence of HBV. Spearman’s correlation analysis suggested that sPD-L1 and sPD-1 levels were positively correlated with HBV DNA level (*r* = 0.23, *P* = 0.011; *r* = 0.26, *P* = 0.004, Fig. 1a). Chi-square analysis showed a significant association between sPD-L1 and HBV DNA level (*P* = 0.008), but no significant association between sPD-1 and HBV DNA level (*P* = 0.12, Table 3). In addition, patients with high serum CRP levels (> 3 mg/L) had significantly higher sPD-L1 levels as than patients with low CRP levels (*P* < 0.01; Fig. 1b). Chi-square analysis also demonstrated a significant association between sPD-L1 and CRP (*P* = 0.023), however, no association between the level of sPD-1 and CRP was identified (*P* = 0.399, Table 3).

**Table 3:** Relationship of sPD-1 and sPD-L1 to patient clinicopathologic features

Variables	sPD1		C*	*P* value	sPD-L1		C*	*P* value
	Low	High			Low	High		
HBV DNA			0.185	0.120			0.272	0.008
0–10^2^	12 (40.0)	27 (30.0)			33 (38.8)	6 (17.1)		
10^2^–10^4^	11 (36.7)	23 (25.6)			26 (30.6)	8 (22.9)		
> 10^4^	7 (23.3)	40 (44.4)			26 (30.6)	21 (60.0)		
CRP (mg/L)			0.077	0.399			0.203	0.023
> 3	24 (80.0)	65 (72.2)			68 (80.0)	21 (60.0)		
0–3	6 (20.0)	25 (27.8)			17 (20.0)	14 (40.0)		
ALT			0.070	0.442			0.131	0.147
≤ 50 U/L	21 (70.0)	56 (62.2)			58 (68.2)	19 (54.3)		
> 50 U/L	9 (30.0)	34 (37.8)			27 (31.8)	16 (45.7)		
AST			0.189	0.035^a^			0.124	0.169
≤ 40 U/L	26 (86.7)	60 (66.7)			64 (75.3)	22 (62.9)		
> 40 U/L	4 (13.3)	30 (33.3)			21 (24.7)	13 (37.1)		
GGT			0.112	0.216			0.215	0.016
≤ 60 U/L	23 (76.7)	58 (64.4)			63 (74.1)	18 (51.4)		
> 60 U/L	7 (23.3)	32 (35.6)			22 (25.9)	17 (48.6)		
Grades of differentiation			0.185	0.120^a^			0.125	0.386^a^
Low	1 (3.3)	16 (17.8)			14 (16.5)	3 (8.6)		
Medium	22 (73.3)	60 (66.7)			58 (68.2)	24 (68.6)		
High	7 (23.3)	14 (15.6)			13 (15.3)	8 (22.9)		
Capsular invasion			0.020	0.825			0.067	0.412
Absent	19 (63.3)	59 (65.6)			57 (67.1)	21 (60.0)		
Present	11 (36.7)	31 (34.4)			28 (32.9)	14 (40.0)		
Microvascular invasion			0.000	1.000^a^			0.151	0.094
Absent	27 (90.0)	81 (90.0)			79 (92.9)	29 (82.9)		
Present	3 (10.0)	9 (10.0)			6 (7.1)	6 (17.1)		

The data represent the numbers of patients, with percentages in parentheses

*AFP* alpha-fetoprotein, *AST* aspartate aminotransferase, *ALT* alanine aminotransferase, *CRP* C-reactive protein, *GGT* gamma-glutamyl transpeptidase

*Pearson contingency coefficient

^a^Fisher’s exact test

mPD-L1/mPD-1 can be upregulated by multiple inflammatory cytokines, such as IL-6, IL-10, IL-12, IL-17, IFN-γ, and TNF-α [18]. However, whether the regulatory mechanisms of sPD-L1/sPD-1 are similar to those of mPD-L1/mPD-1 remains unknown. Therefore, we subsequently investigated a series of inflammatory cytokines (as described in the “Materials and methods” section) to identify the significant cytokines associated with sPD-L1/sPD-1 levels. Our results showed that high serum levels of IL-10, IL-17 and TNF-α correlated with high levels of sPD-L1 and sPD-1 in HCC patients. Intriguing, high serum IFN-γ levels correlated with high sPD-1 levels, while no association between IFN-γ and sPD-L1 was identified (Table 4). In addition, associations between other inflammatory cytokines and sPD-L1/sPD-1 were not detected (data not shown).

**Table 4:** Associations of soluble PD-1/PD-L1 and serum markers with tumor IHC

Variables	sPD-L1		*P* value	sPD-1		*P* value
	Low	High		Low	High	
Serum markers (median, range)						
IFN-γ pg/mL	828.2 (0–5252.7)	1044.6 (198.5–4899.4)	0.170	539.3 (0–2657.4)	1046.1 (0–5252.7)	0.012
IL-10 pg/mL	68.4 (0–3172.9)	126.7 (21.2–2255.1)	0.009	43.4 (0–1038.8)	109.4 (0–3172.9)	0.003
IL-17 pg/mL	0.8 (0–29.5)	1.7 (0–42.2)	0.006	0.5 (0–5.3)	1.1 (0–42.2)	0.005
TNF-α pg/mL	131.7 (8.9–890.5)	193.2 (29.4–1062.2)	0.038	104.1 (10.5–890.5)	177.1 (8.9–1062.2)	0.030
Tumor IHC						
PD-L1 expression			0.660			0.399
Low	64 (75.3)	25 (71.4)		24 (80.0)	65 (72.2)	
High	21 (24.7)	10 (28.6)		6 (20.0)	25 (27.8)	
CD4^+^ TILs			0.349			0.455
Low	48 (56.5)	23 (65.7)		19 (63.3)	50 (55.6)	
High	37 (43.5)	12 (34.3)		11 (36.7)	40 (44.4)	
CD8^+^ TILs			0.722			0.335
Low	48 (56.5)	21 (60.0)		20 (66.7)	51 (56.7)	
High	37 (43.5)	14 (40.0)		10 (33.3)	39 (43.3)	

The Wilcoxon–Mann–Whitney test and Chi-square test were used to compare continuous and categorical variables between groups, respectively

### Associations between sPD-1/sPD-L1 and other clinicopathological characteristics

The associations between other clinicopathologic characteristics and sPD-L1/sPD-1 were further assessed (Tables 1, 3). High levels of sPD-1 tended to be found in patients with higher baseline AST levels, and high sPD-L1 levels were prevalent in patients with high levels of GGT. The associations between sPD-L1/sPD-1 and AFP level, Child-Pugh score, tumor size, HBV history, BCLC stage, tumor number, grade of differentiation, microvascular invasion and capsular invasion were insignificant.

### Associations between Intratumoral PD-L1, tumor-infiltrating lymphocytes and sPD-L1/sPD-1 levels

Based on previous reports [11], sPD-L1/sPD-1 expression may closely correlate with CD4/CD8^+^ TILs. Therefore, we conducted immunohistochemical staining for CD4 and CD8. In addition, we also analyzed intra-tumoral expression of PD-L1 and Foxp3^+^ TILs. Representative staining images of PD-L1, CD4, CD8 and Foxp3 are shown in Supplementary Figs. 2 and 3. PD-L1 positive staining was predominantly observed on tumor cell membranes and on some TILs (Supplementary Fig. 2a). No significant associations of intra-tumoral expression of PD-L1 with OS and DFS were determined (Table 2). Meanwhile, there is no obvious difference in DFS (*P* = 0.521) and OS (*P* = 0.081) between the high and low number of Foxp3^+^ TILs patients (Supplementary Fig. 1a and b). In addition, we did not find any associations between sPD-L1/sPD-1 and either intra-tumor al expression of PD-L1 or the numbers of CD4^+^ TIL and CD8^+^ TILs.

## Discussion

An increasing number of studies have reported that sPD-1 and sPD-L1 might play crucial roles in the prediction of treatment responses and prognosis in cancer patients [19–21]. However, the regulation, source and prognostic value of sPD-1/sPD-L1, as well as their association with clinicopathological factors in HCC, remain matters of debate.

To date, there is no consensus on the cut-off values for sPD-1/sPD-L1 in cancer patients. Therefore, our study used the X-tile software program to determine the best cut-off values of sPD-1 and sPD-L1 for predicting DFS (33.0 µg/mL and 11.2 µg/mL, respectively). In multivariable analysis using a Cox regression model, we found that sPD-L1 was a negative independent prognostic factor [Hazard Ratio (HR) for DFS 2.58 (1.14–5.84), *P* = 0.023; HR for OS 1.77 (1.01–3.12), *P* = 0.048] in accordance with a recent study [14], while sPD-1 was a favorable independent prognostic factor [HR for DFS 0.32 (0.14–0.74), *P* = 0.007; HR for OS: 0.54 (0.30–0.98), *P* = 0.044] in HCC. For other malignancies, such as lung cancer [19] and diffuse large B-cell lymphoma [9], several reports have also demonstrated that sPD-L1 is an unfavorable prognostic biomarker. Regarding sPD-1, an elevated sPD-1 level was associated with prolonged OS (*P* = 0.006) and progression-free survival (*P* = 0.013) for patients with non-small cell lung cancer undergoing erlotinib therapy [12]. In fact, in a previous study of murine HCC, delivery of sPD-1 into tumor site using adeno-associated virus resulted in enhancement of antitumor immune effects and ultimately reduced tumor growth and prolonged long-term survival [22]. Preclinical studies indicated that sPD-1 is bioactive, which could counteract the immunosuppressive effect of the PD-1/PD-L1 pathway, leading to restored T-cell function, a decreased number of regulatory T cells and enhanced antitumor immunity [23, 24]. These earlier findings may explain why the elevated level of sPD-1 seems to play a favorable role in prolonging DFS and OS in patients with HCC.

In virus-associated malignancy, mPD-1/mPD-L1 are upregulated in the context of persistent inflammation caused by the presence of the virus [25–27]. We hypothesized that the regulatory mechanisms of sPD-1/sPD-L1 might be similar to those of mPD-L1/mPD-1 in patients with HCC. Accordingly, we found that sPD-L1 and sPD-1 were positively correlated with HBV viral load and sPD-L1 was also associated with a systemic inflammatory marker (CRP level), which suggested that sPD-L1 and sPD-1 can also be induced by persistent inflammation caused by HBV infection. An increasing number of studies have demonstrated the existence of T-cell exhaustion in virus-associated malignancies, including HBV related HCC [25–28]. Therefore, blockade of the PD-1/PD-L1 pathway could enhance the anti-tumor immune response and facilitate the restoration of virus-specific T cells in HCC.

mPD-L1/mPD-1 can be induced by multiple inflammatory cytokines in cancer, including IFN-γ, IL-6, IL-10, IL-17 and TNF-α (5, 17, 29, 30). However, the presence of similar regulatory mechanisms between inflammatory cytokines and sPD-L1/sPD-1 in cancer remains unknown. Therefore, we subsequently tested a series of inflammatory cytokines to identify their associations with sPD-1/sPD-L1. Antibody array assays, showed that high serum levels of IL-10, IL-17 and TNF-α correlated with high levels of both sPD-L1 and sPD-1, while it is intriguing that high serum levels of IFN-γ were correlated only with high sPD-1 levels. While we discovered an association between certain inflammatory cytokines (IL-10, IL-17, TNF-α and IFN-γ) and sPD-1/sPD-L1, whether or not these inflammatory cytokines are involved in inducing the levels of sPD-L1/sPD-1 needs further investigation. In fact, sPD-L1 and sPD-1 were initially described in autoimmune disease, where both sPD-L1 and sPD-1 were thought to be induced by similar inflammatory cytokines [29, 30]. We also observed some similar associations between inflammatory cytokines (IL-10, IL-17, TNF-α) and sPD-1 or sPD-L1, as well as a close positive association between sPD-1 and sPD-L1. Our results were partly consistent with those of previous studies in autoimmune diseases [29–31], which might indicate a similar origin or regulation of sPD-1 and sPD-L1 in HCC patients.

In addition to sPD-1/sPD-L1, we also analyzed the intra-tumoral expression of mPD-L1 and the number of TILs in HCC patients. A total of 89 (74.2%) patients had low mPD-L1 expression, and 31 (25.8%) had high mPD-L1 expression. The associations between mPD-L1 expression and DFS or OS were insignificant in both univariate and multivariable analysis, which was partly in line with the results of previous study [7]. As reported recently, in pancreatic cancer patients, the association between sPD-L1 level and intra-tumoral expression of PD-L1 was found to be insignificant [11]. Similarly, the intra-tumoral expression of PD-L1 did not associate with sPD-L1 levels in our study. In addition, it has been reported that TILs were a significant prognostic factor and seemed to be related to PD-L1 expression in cancer [3, 11], hence, we subsequently explored their prognostic values and associations with sPD-1/sPD-L1 expression. The number of CD8^+^ TILs was an independent prognostic factor for OS in our study. However, no significant associations were identified between TILs and sPD-1 or sPD-L1. These conflicting results may be due to the fact that the majority (57%) of tumor samples in the previous study [11] were from metastatic sites and the number of TILs and tumor microenvironment might differ between primary tumors and metastases in HCC, as has been described in colorectal cancer [32] and breast cancer [33].

We also conducted an additional analysis of DFS and OS in HCC broken down into eight subgroups by levels of IFN-γ, sPD-1 and CD8^+^ TILs (Supplementary Fig. 4). Interestingly, the survival curves showed that patients with high sPD-1 and high IFN-γ levels and high CD8^+^ TILs had better survival than other subgroups, apart from the subgroup with low sPD-1 and low IFN-γ levels and high CD8^+^ TILs. Currently, due to the limited sample size of this study, subdividing the population into eight subgroups precludes solid inference. These interesting results may prompt us to do more further exploration in our subsequent studies.

Although our study identified the significant prognostic value of sPD-1 and sPD-L1 levels in HCC patients, several limitations of our research remain. First, the data might be subject to selection bias due to the single-center retrospective design of this study. Second, the specimen bank of our hospital only contained serum samples from the patients’ first admission. As the immune system in each patient is dynamically changing, monitoring the changes in the level of serum sPD-1/sPD-L1 might provide insight into the regulation and immunologic function of sPD-1/ sPD-L1 in HCC.

In conclusion, our study demonstrates that sPD-1 and sPD-L1 are independent prognostic biomarkers with opposite prognostic roles in HCC patients. The similar associations between inflammatory cytokines (IL-10, IL-17 and TNF-α) and both sPD-1 and sPD-L1, as well as the positive association between sPD-1 and sPD-L1 levels, suggest a similar origin or regulation of sPD-1 and sPD-L1 in patients with HCC.

### Electronic supplementary material

Below is the link to the electronic supplementary material.

Supplementary material 1 (PDF 645 KB)

## Abbreviations

AFP: Alpha-fetoprotein
ALT: Alanine aminotransferase
AST: Aspartate aminotransferase
BCLC: Barcelona clinic liver cancer
CRP: C-reactive protein
DFS: Disease-free survival
GGT: Gamma-glutamyl transpeptidase
HCC: Hepatocellular carcinoma
HR: Hazard ratio
mPD-1: Membrane-bound PD-1
mPD-L1: Membrane-bound PD-L1
PD-1: Programmed death protein 1
PD-L1: Programmed death ligand 1
sPD-1: Soluble PD-1
sPD-L1: Soluble PD-L1
TILs: Tumor-infiltrating lymphocytes
