# Supplementary material for: The correlation and prognostic value of serum levels of soluble programmed death protein 1 (sPD-1) and soluble programmed death-ligand 1 (sPD-L1) in patients with hepatocellular carcinoma
Source: Cancer Immunol Immunother. 2018 Dec 1;68(3):353–63. doi: 10.1007/s00262-018-2271-4 (PMC6426820; doi:10.1007/s00262-018-2271-4)
Supplement: Supplementary file 1 — Supplementary material 1 (PDF 645 KB) [file 262_2018_2271_MOESM1_ESM.pdf]

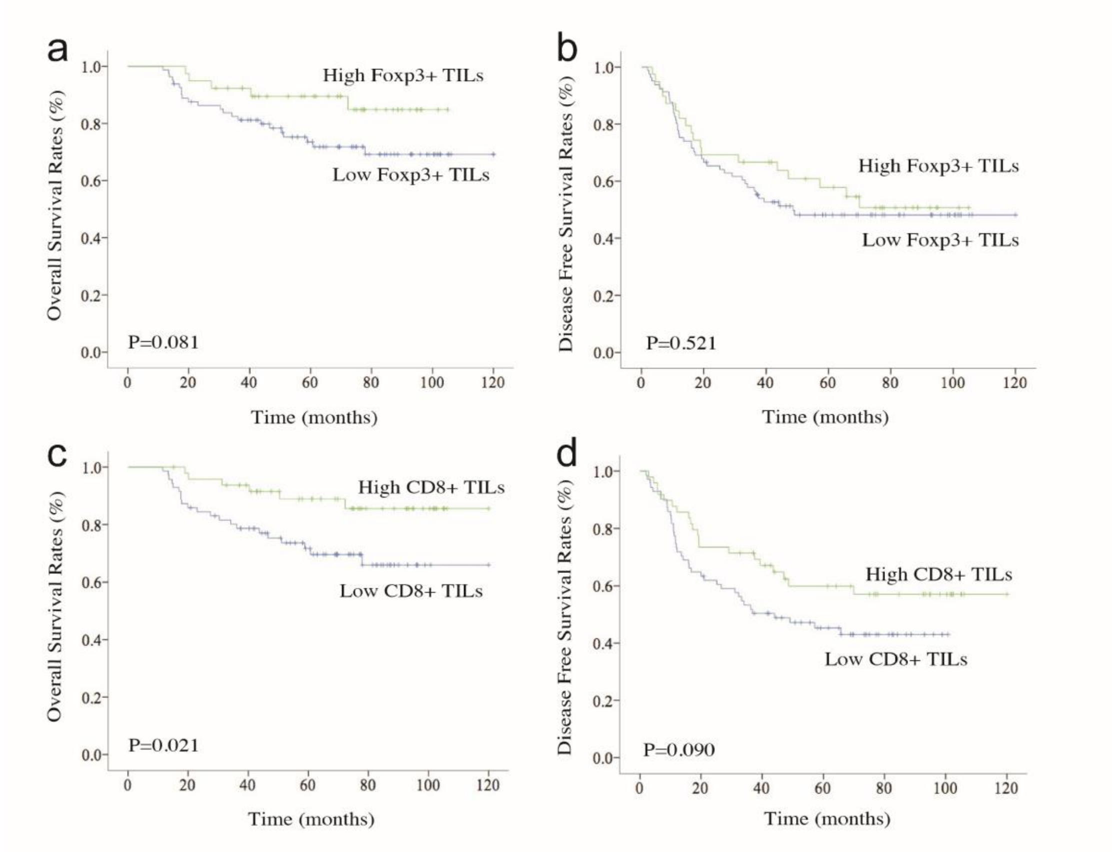

Supplementary Figure 1. The effects of the number of Foxp3$^+$ TILs and CD8$^+$ TILs on prognosis.

Kaplan-Meier survival curve for overall survival (OS, a) and disease-free survival (DFS, b) according to the number of Foxp3$^+$ TILs as well as the OS (c) and DFS (d) by the number of CD8$^+$ TILs.

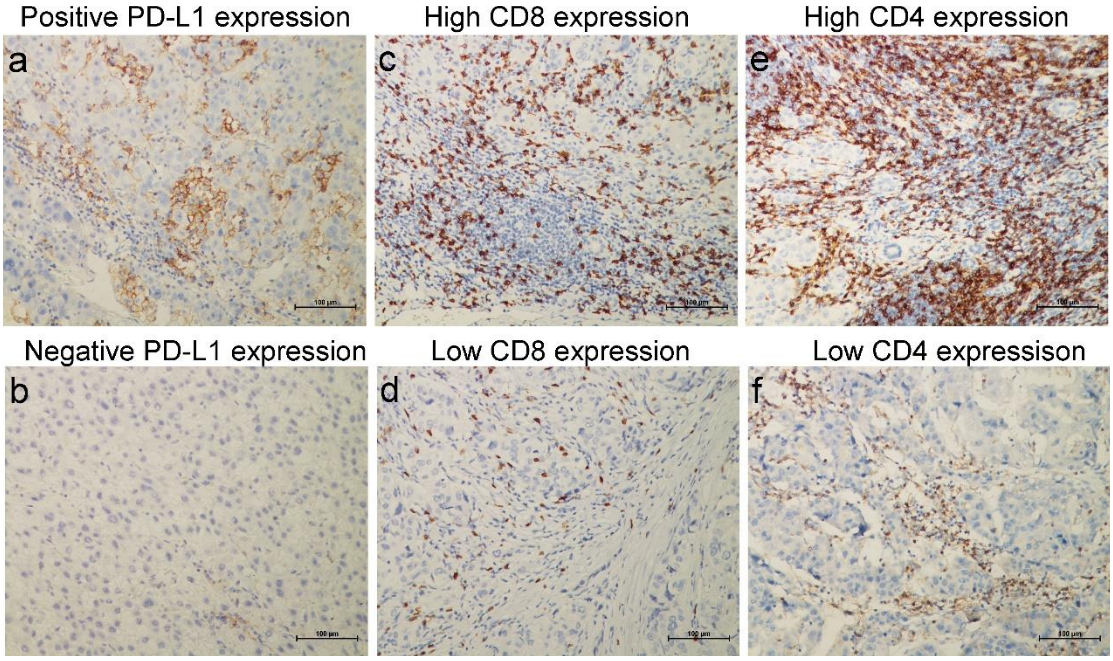

Supplementary Figure 2. Immunohistochemical staining of PD-L1, CD4 and CD8 in HCC.

(a) PD-L1-positive tumor cells (PD-L1 expression ≥5 %); (b) PD-L1-negative tumor cells (PD-L1 expression <5 %); (c) $CD8^+$-high tumor infiltrating lymphocytes; (d) $CD8^+$-low tumor infiltrating lymphocytes; (e) $CD4^+$-high tumor infiltrating lymphocytes (f) $CD4^+$-low tumor infiltrating lymphocytes; (a - f, 200× magnification).

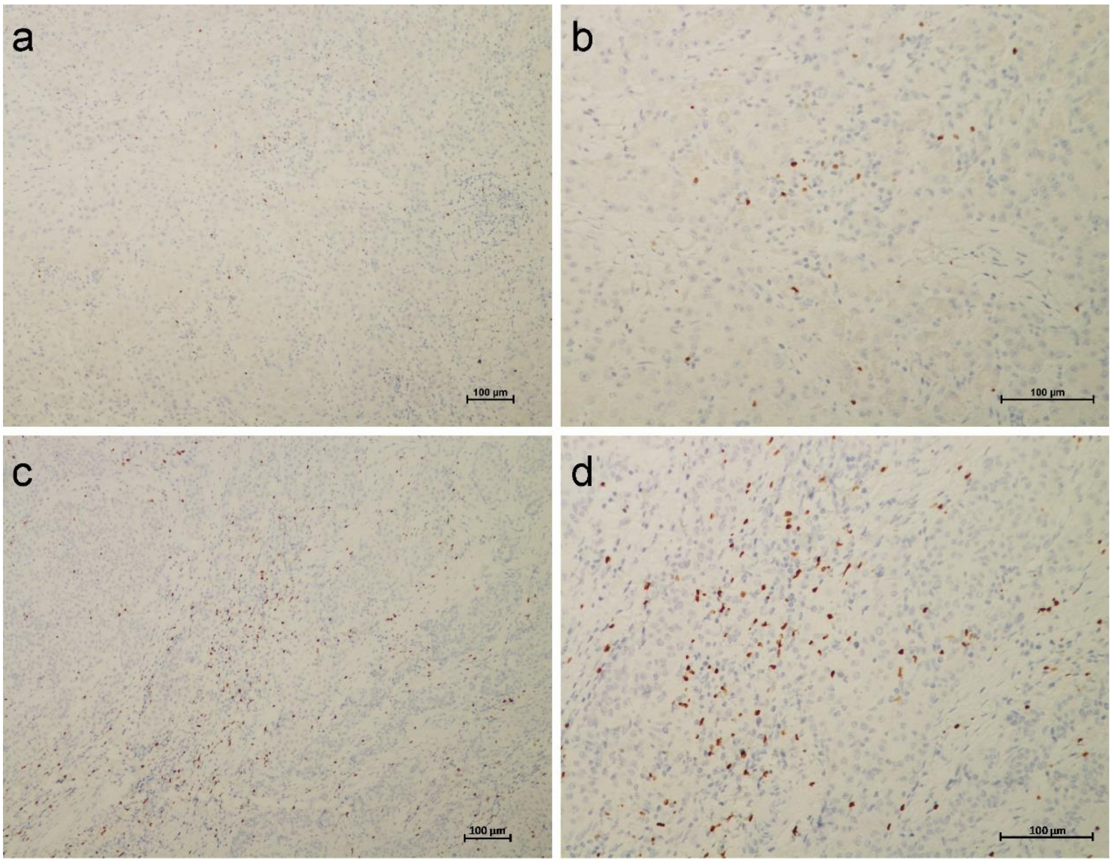

Supplementary Figure 3. Immunohistochemical features of Foxp3 expression in HCC.

(a, b) A low number of Foxp3$^+$ tumor infiltrating lymphocytes; (c, d) A high number of Foxp3$^+$ tumor infiltrating lymphocytes; (a and c, 100× magnification; b and d, 200× magnification).

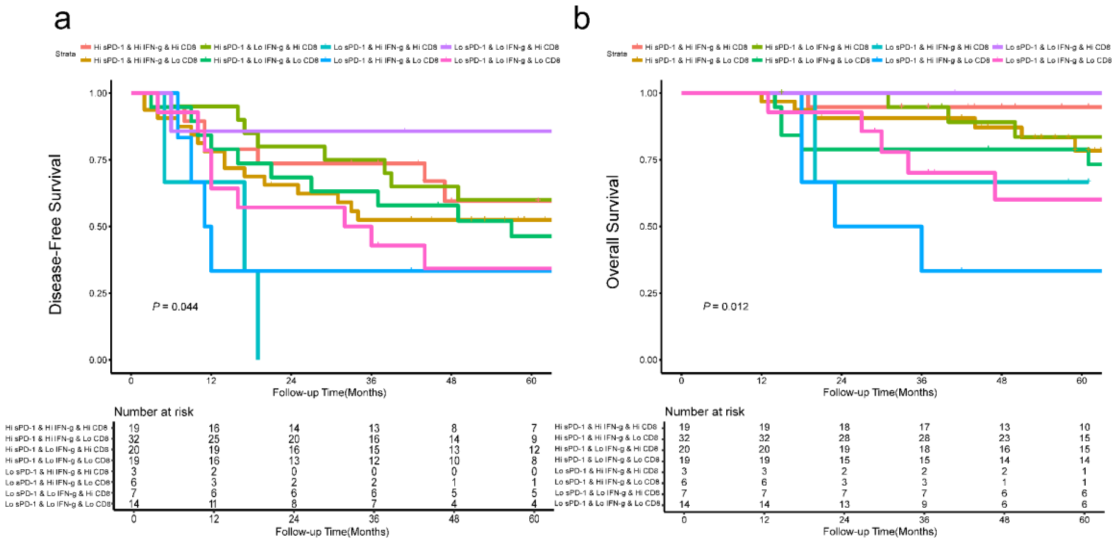

Supplementary Figure 4. Kaplan–Meier estimates of OS and DFS in HCC patients broken down into eight subgroups by levels of IFN-γ, sPD-1 and CD8$^+$ TILs.

Kaplan-Meier curves for OS (a) and DFS (b) according to subdivision by the levels of sPD-1 and IFN-γ, as well as the number of CD8$^+$ TILs. Lo, low; Hi, high.

Supplementary Table 1. Kappa values for the inter-rater reliability for each IHC staining factor.

IHC staining factor	Kappa Value
PD-L1	0.898
CD4	0.832
CD8	0.841
Foxp3	0.812
